# Supplementary material for: Patterns of Intron Gain and Loss in Fungi
Source: PLoS Biol. 2004 Nov 30;2(12):e422. doi: 10.1371/journal.pbio.0020422 (PMC532390; doi:10.1371/journal.pbio.0020422)
Supplement: Table S1 — Also available at http://genes.mit.edu/NielsenEtAl/. (4.3 MB ZIP). [file pbio.0020422.st001.zip › NielsenEtAl/html/679.html]

AN6889.1.NCU00445.1.MG05205.1.FG04109.1


```
 CLUSTAL W (1.82) Multiple Sequence Alignments - Introns Inserted


Sequence 1: NCU00445.1	1115 aa
Sequence 2: FG04109.1	1013 aa
Sequence 3: MG05205.1	1167 aa
Sequence 4: AN6889.1	1046 aa
Alignment Length: 1241 aa
Number Identitical Residues: 290 aa
Alignment Score (without introns) 17304


MG05205.1 	MAQYAVKSVDDWATHNTATEAGATANSTTAADASRDRESVSATVQELESDLVGKSTAVTT
NCU00445.1	MAQYAGS----------------AAQQAAAGHQSLSPLSPSASVSASVGTAPPLTPSGTA
FG04109.1 	MAPYG-------------------ANQPTSG-----------------------------
AN6889.1  	------------------------------------------------------------
          	                                                            

MG05205.1 	ATPINDNINRASKTTTTTTTTAAATDDDAYRARSRSPVTGKPKPNAPSSPATHSQTAPAA
NCU00445.1	PAGT----------------------------------------AAAAGPAPSAATAQSG
FG04109.1 	--------------------------------------------ASDSSPAASINNATQG
AN6889.1  	-------------------------------------------MATHDGQQDNGLYSRET
          	                                             :  .       :   

MG05205.1 	RQQRHQQNPSSNSADHSSASPATSAQAPGTASAAGQQPQQHRRVYQACI1PCRRRKVRCD
NCU00445.1	AGSSSASQPQDKPRSQSQSGAGPSAAGGGASSTTGAQ---HKRVYQACI~PCRRRKVRCD
FG04109.1 	SAQQTPGQ----------------SAG---NANQGPQ---HKRVYQACI~PCRRRKVRCD
AN6889.1  	SARRSPSP-------------------------TGPQ---HRRGYQACD~PCRKRKVKCD
          	                                  * *   *:* ****  ***:***:**

MG05205.1 	LGS~VDNPHDPPCVRCRRESKECYFSATRRKRKADDED----LSGAEDDYVLRNGRKRVH
NCU00445.1	LGS~VDNPSDPPCVRCRRESKECYFSATRRKRKVDDDMTDIFDDPDHDDYILRNGRKQLR
FG04109.1 	LGS~VDNPHDPPCVRCRRESKECFFSATRRKRKTDD------DGSDQDEYIIRNGRKRVN
AN6889.1  	LGS1VDNPRPPPCVRCRRESKRCEFSATRRKRKTSDVEGSAVDDVLRRDKRMMVGDAAAK
          	*** ****  ***********.* *********..*   .   .  . :  :  *    .

MG05205.1 	EGSPP--------IDRRMYSDVPLTPGGSQGRTQPLTRPRHGSVGNDGKRTGRASGAGLN
NCU00445.1	SGESPV---MAAPLDRRLYSDVPLTPGGSRGRAQPLRRPGDGGPNSTTSTGGARSTTGGE
FG04109.1 	ANDSP-----PPLIERRSYSSAPLTPGGSHGQAQPLRRP-DG-----SRIGRGRNSDWG-
AN6889.1  	IEASPSERSASYPQVEPSFDTPNLSQQKWLNKSPSNGHLPAPSNGSQHYTANPSTSTAQF
          	   .* . : .    .  :.   *:     .:: .  :    . ..        .     

MG05205.1 	SQTDAPNTPLENMEARTLMRREVYGPHDALDLLYKAATDS2PHKLGEAEANSRTSQTAAA
NCU00445.1	FGAGEANTPLENLEARTVMRREVYGPHDALDLLYKAATDK~LVDTRSDRSQRLPYADVAR
FG04109.1 	-TEGDANQTLENIEAQTVMRRGVFGPQDALDLLYKAATDS2PAVERERR-----------
AN6889.1  	SDVRNTRHPAYSAGDRTSVSRFSLEGNQPMMNRTAVELLS~PAISNSHDALHLLSEAAGR
          	     .. .  .   :* : *     ::.:     .   .      .  :        . 

MG05205.1 	ASVNFRVPAAESAARPVSARDIG----RRQSSFRAESRQSGSQ-QNRRTAARPEAPPVDA
NCU00445.1	LRSPMHRQDGESTAAGDSSRTNHHAG-RPGLNSRHSSHHNDRPGYHSRHASRQEA--STE
FG04109.1 	----------ESTSSARPAAP------------RPPPVDTGPYGYVPRSTSRPPK--TEQ
AN6889.1  	TEDLRYAATRQSASSFASPISSMTQAGTPRSTGGSFSRQHRSGTAQTGNYYQSNALLSGE
          	          :*::   ..      ..    .    . .            :        

MG05205.1 	PIDPQLTAKRDVTSDPGYTDALKAWARFRFVRAGWFTAHEAIEYIDY2YYHYLSPLTPIS
NCU00445.1	SIDPELT-KRDISQEPGYNEALKAWARFRFVRAGWFTAQEAIDYIEY2YYAYLSPLTPIS
FG04109.1 	PIDPELS-RPELSSQPGYAEAIKAWQRFRFVRAGWFTALEAIEYIDY2YYKYLSPLTPIS
AN6889.1  	SLADNRTSSTGRPSDSGYLDAVKAWSRLRFVRAGWLSVEESMAYVAY2YYEHLAPLSPIV
          	.:  : ::    ..:.** :*:*** *:*******::. *:: *: * ** :*:**:** 

MG05205.1 	PPTFKSPSTHLTLLTEEPILTVTLLTIASRYRQVPGTGGRCRSQAIHEQLWTYLRGMIER
NCU00445.1	PPTFRNPASHLTLLTEEPILTVTLLTIASRYRRMPGTGGHCRSHAIHEQLWTYLRGMIER
FG04109.1 	PPTFSNPSSHVTLLYEEPILTVTLLTIASRYRQMPGTGGHCRSHAIHEQLWMYLRGMIER
AN6889.1  	IPDFSHPSTHRTLLTDEPVLAVTILTTASRHMKPSGDGANSRAFYIHDRLWSYLRGMIER
          	 * *  *::* *** :**:*:**:** ***: : .* *...*:  **::** ********

MG05205.1 	VVWGQEAFGGGFCGSYTMG--HEEAQTSSTAPWRGLRKGSLRTLGTIESLLILTEWHPRA
NCU00445.1	VVWGQEAFGGGFCGSG-----ADESQSSSTAPWRGLRRGSLRTFGTIESLMILTEWHPRA
FG04109.1 	CLWGQEAFGGGFCVPPSTSAIFDESQTSSTAPWRGLRKGSLRTLGTIESLMILTEWHPRA
AN6889.1  	LFWGQEKFGGNGIGIN-----KPRSFDLAPSSAKVNHKGNLRSLGTIEALLILTDWHPRN
          	 .**** ***.            .:   :.:. :  ::*.**::****:*:***:**** 

MG05205.1 	LHFPPAEAIDELMLPSLYDGPDEHMGPDG---AAQRPSATTGVGGKRIESWLEPAWRSDR
NCU00445.1	LHFPPSEAIDELLLPSYEHN----SADDD---NNQKPSA--GIGGRRIESWLEPAWRSDR
FG04109.1 	LHFPPTEATDELVLPMEAAFQAITAADED---PSKTLGP--GFGGKRIESWLEPAWRSDR
AN6889.1  	LHFPPGDDENALLDLDAQAGRYDKELDNDGETTAQRSSSGAPEGRLAFQTWLEPAWRSDR
          	***** :  : *:              :...:  :  .. :  *   :::**********

MG05205.1 	MCWMLLSTAMGLAYELGVFDDIEELLATGAIARPEYEDEAYRLRAHRIKRLVLVYLSQLA
NCU00445.1	MCWMLLSTALGLAYELGVFDEIDELLATGSITRPEYEDESYRLRAHRIKRLLLIYLSQLA
FG04109.1 	MCWMLLSTANGLAYELGVFDDIDELLRDDAITRPEYQEESYRQRAFRIKRLLLIYTTQLA
AN6889.1  	MSWMLLSTAQALAFELGVFDQKND-------TKLSAEPPAEQTRKRRLRRLILVYITQSS
          	*.******* .**:******: ::       :: . :  : : *  *::**:*:* :* :

MG05205.1 	GRLGWTNMVPENLRKSDSVLAPRRPAS-EGATPGTTLSAISGSFNYIPDLEMDDQIIHCW
NCU00445.1	GRLNWTNMVPENMR-NDPIMSRRRPHSGEGTTPGTNPSSTSNGFVYIPDLELDDQIILCW
FG04109.1 	GRLGWTNMAPAHLRRSDPALARFRPNTGDGTTPGTNPSSVGNNFNYVPDLELDDQIIHCW
AN6889.1  	GRLGIPSMLPLPQWTDDIQP-----------TPLTGVKGNE-----------VDKMHDCW
          	***. ..* *     .*              ** *  ..              *::  **

MG05205.1 	AGISHAMYMGNEKLFRSRKHTTDIIQSGRYIQLLEDFTPMLREWWRQFERYRL~PPYIRH
NCU00445.1	AGVSNVMHRGNEKLFRSRNYTTDIIQSGKYIQLLKEFQPLLRNWWNDFNRFVL~PPYIRH
FG04109.1 	AGISNAMEMGNEKIFRSRKHTTQIIQNGKYISILKEFEPILRDWWREFELFRL~PEFIRH
AN6889.1  	LGISKIMYQSNQLLFASNEQTSDLIRSGRYRDQIDRFQPFLREWRQNIDSTEL1HPAMRH
          	 *:*: *  .*: :* *.: *:::*:.*:* . :. * *:**:* .:::   *    :**

MG05205.1 	ILTIEYEYVR~IYINSLSLQAVVERCTN------------NASNASGHGNDQLSPQTQNY
NCU00445.1	ILTIEYEYVR~IYVNSLALQAVVERCTNNPNVGGGGST--NGGSVSGHSAPQLSPQTQNY
FG04109.1 	ILTIEYQYVR~IYINSLSLQAVVERCTNQAQAGATAHSGHHGAAAVNGGHPQLSPQTMIN
AN6889.1  	ILMIEYEYTR1LYVNSLALQAVVDRWTT------------MSNEAAQAQNKPSASNNASF
          	** ***:*.* :*:***:*****:* *.             .  .        :.:.   

MG05205.1 	FGKLPLGHLGGFGVQDQDYVKEVVDGSRNLLRAVVDGLLPNDYLKHAPVRTYFRIISGAM
NCU00445.1	LGKLPLSQLGGFGAEDEEYVKEVVNGSRNLLRTVVDGLLPKGYLKHAPVRTYFRIISGAM
FG04109.1 	YGKLPLGQLGGFTAHDQEYIREVVEGSRNLLRTVVEGLLPGDYLKHAPVRTYFRIISGAM
AN6889.1  	HVLMELYRVN------EPFIQEVVDASRRILTTVLEGLVPGDHLKHAPVRTCFRILSGMI
          	   : * ::.      : :::***:.**.:* :*::**:* .:******** ***:** :

MG05205.1 	FLLK~TFALGAPRSDVEISIALMDRTVDALRNCVVDDVHLGIRFADLLETLTSRLRNRFI
NCU00445.1	FLLK~TFALGAPKSDVELSISLMDQTVDALRHCVVDDVHLGIRFADMLETLTSRLRNRFI
FG04109.1 	FLLK~TFALGAPRSDVRMSIELMDATVEALRNCVVDDVHLGIRFADLLESLTSRLRNRFI
AN6889.1  	FILK0TFTLGAKEDDVRVSLDLQDRTVEALRTCVVDDIHLSHAIARLLELLTTNIRTRFL
          	*:** **:*** ..**.:*: * * **:*** *****:**.  :* :** **:.:*.**:

MG05205.1 	HAPTHTLNNHPIPGGSSTDRLSPQPRVDSGMADTNMGGMHISSHDVDTWLRPNGQGSLAS
NCU00445.1	HAPP--------PPVATTDGRSPNP---NHQADTTNG-NGPMPHGAPNLSSGD---VWSK
FG04109.1 	QAPT--------MQQASGRGQSPAP---DGQVHNGEN-LVPSGEDNPNWVGGHAQRLRDG
AN6889.1  	RFAPLDRSGDN-DSTSAGQDRASAPTSRAHSPRSREGPLGRRDGLNNSHTWPSAQSTHNN
          	: ..   ..      ::    :. *        .  .          .     ..     

MG05205.1 	LS---AAA~AAVANPHGLD~RSTTPANISATPFEISAGNI----PYPTGQASIFAPTTPA
NCU00445.1	LS---ANN~--------LE1RGNPTNNISATPWDVAAGDF----PYPTGSASMFGPSTPA
FG04109.1 	LN---GND1PAPA----QA~ATHEANNISATPFDLSAQNF----PYPGQGP--LGPSTPT
AN6889.1  	QIGGYADA~HPPSSTPLTS~VHDPLAGIPAQPINSSNINVNFMPPPPSVYYNFYQPRSPP
          	  .. .    . :..           .*.* * : :  :..  .* *    .   * :*.

MG05205.1 	ASGSMDHHHN--------------PLPDNMFGDPSDWNNSSSEMWYLPTGPAFFQNMGDS
NCU00445.1	ATSNLPDTNGNSNGNGGGGGGGGGGLTDSMFEN-TDWANPSNEMWYLPTGPAFFQNIDNT
FG04109.1 	VVDNAVAN-----------------MDVSLFE---DWNHQGNEMWYLPPGPAFFQNVENP
AN6889.1  	PSGEMNPSNPNS-----------GSASSNLPSHSMNEQPGVSDWFALPLDQFFNSSTAVV
          	  ..      .:           .    .:   . :     .: : ** .  * ..    

MG05205.1 	AVAMTAEGVNVGGIDLLEYMAMDQF----PIEGSGF--------------
NCU00445.1	SVSMTAEGVNVGGVDLLDFMAMD------PMDGASFVLDGGRYP------
FG04109.1 	SVAMGPEGVNVGGLDLLEYMAMD------PSQFPGLDPSSGPPTSHA---
AN6889.1  	DQGLGGTGPMVGEFDMLEVLLNEQYDGGTSGDGTGLDSAGGGLPSQFIQP
          	  .:   *  ** .*:*: :  :. ...:. : ..:   ..  .:   ..
```
